# Enabling Broadband Solar‐Blind UV Photodetection by a Rare‐Earth Doped Oxyfluoride Transparent Glass‐Ceramic

**DOI:** 10.1002/advs.202309433

**Published:** 2024-01-15

**Authors:** Hong Jia, Rui Zhang, Xuying Niu, Xian Zhang, Hui Zhou, Xiaofeng Liu, Zaijin Fang, Fei Chang, Bai‐Ou Guan, Jianrong Qiu

**Affiliations:** ^1^ College of Physics and Electronic Information & Henan Key Laboratory of Electromagnetic Transformation and Detection Luoyang Normal University Luoyang 471934 China; ^2^ Longmen Laboratory of Luoyang Luoyang 471000 China; ^3^ Department of Optoelectronics Science Harbin Institute of Technology Weihai 264209 China; ^4^ School of Materials Science and Engineering Zhejiang University Hangzhou 310027 China; ^5^ Guangdong Provincial Key Laboratory of Optical Fiber Sensing and Communications Institute of Photonics Technology Jinan University Guangzhou 511443 China; ^6^ Senba Sensing Technology Co., Ltd. NanYang 473300 China; ^7^ College of Optical Science and Engineering Zhejiang University Hangzhou 310027 China

**Keywords:** down‐conversion, fluoride glass, solar‐blind UV photoelectric detection, Tb^3+^ ions

## Abstract

Oxyfluoride transparent glass‐ceramics (GC) are widely used as the matrix for rare‐earth (RE) ions due to their unique properties such as low phonon energy, high transmittance, and high solubility for RE ions. Tb^3+^ doped oxyfluoride glasses exhibit a large absorption cross section for ultraviolet (UV) excitation, high stability, high photoluminescence quantum efficiency, and sensitive spectral conversion characteristics, making them promising candidate materials for use as the spectral converter in UV photodetectors. Herein, a Tb^3+^ doped oxyfluoride GC is developed by using the melt‐quenching method, and the microstructure and optical properties of the GC sample are carefully investigated. By combining with a Si‐based photo‐resistor,a solar‐blind UV detector is fabricated, which exhibits a significant photoelectric response with a broad detection range from 188 to 400 nm. The results indicate that the designed UV photodetector is of great significance for the development of solar‐blind UV detectors.

## Graphical abstract

A Tb^3+^ ion doped glass‐ceramic (GC) obtained by the traditional melt‐quenching method, is employed as the converter to combine with a silicon photo‐resistor for the development of a solar‐blind UV detector. Due to the efficient conversion of broadband solar‐blind UV light (188–400 nm) into visible light by the GC, the device exhibits meaningful photovoltage response at a low bias voltage. The research results of this work are of great significance for the development of efficient broadband solar‐blind UV detectors at a low cost.

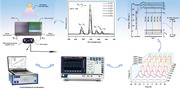

## 1 Introduction

At present, the detection of infrared and ultraviolet (UV) bands other than visible light has gained growing interest. Solar‐blind UV light, that is, far UV and vacuum UV light with a wavelength of less than 0.28 µm, is absorbed by atmospheric ozone and water vapor and cannot reach the Earth's surface.^[^ ^1^, ^2^, ^3^, ^4^, ^5^, ^6^, ^7^, ^8^, ^9^, ^10^, ^11^, ^12^, ^13^ ^]^ The background noise of the solar‐blind UV is small, and it is almost not interfered by solar radiation in the near‐surface space. Therefore, the photodetector working in this spectral range has the advantages of low noise, anti‐interference, and high sensitivity, and has a wide range of application value in military, communications, and civilian fields such as flame sensing, UV communication, ozone detection, and missile attack warning.^[^ ^14^, ^15^, ^16^, ^17^, ^18^, ^19^, ^20^, ^21^, ^22^, ^23^, ^24^, ^25^, ^26^ ^]^ Therefore, UV detection, especially solar‐blind UV detection technology, is a dual‐use detection technology for military and civilian purposes and is also a very useful detection technology for near‐surface space research.

Selecting efficient detector core materials is the key to developing solar‐blind UV detector applications. In previous studies, the spectral converter used for solar blind UV detection was usually based on crystalline luminescent materials in the form of powders and nanoparticles.^[^ ^13^, ^24^, ^25^ ^]^ The development of a transparent and monolithic medium with efficient UV‐excited photoluminescence is of high significance for photodetector applications. Oxyfluoride transparent glass ceramics (GC) feature unique characteristics of low phonon energy, efficient luminescence, and high conversion efficiency of rare earth (RE) ions due to the precipitation of fluoride nanocrystals. Moreover, oxyfluoride GCs possess excellent features of glass, such as high optical transmittance, high thermodynamic stability, and easy fabrication for bulk samples and fibers, which is hardly achieved by crystals. These benefits make RE‐doped oxyfluoride GC a candidate material for laser and detection applications in the ultraviolet (UV) and vacuum ultraviolet (VUV) wavelength range. In addition, it offers particular advantages including high mechanical strength, chemical durability, and thermal stability of oxidized glass.^[^ ^27^, ^28^, ^29^, ^30^, ^31^, ^32^, ^33^, ^34^ ^]^ There have been several studies on the spectroscopic properties of oxyfluoride GC doped with RE ions like Sm^3+^, Dy^3+^, Gd^3+^, and Tb^3+^. Among these systems, Tb^3+^ doped GCs exhibit strong and efficient visible emission under UV excitation. Because the 4f orbital of Tb^3+^ ions is not in a semi‐full state and is extremely unstable, the ^5^D_4_→^7^F_6_ electronic transition is highly efficient due to the presence of a more stable ^4^F_7_ energy state. The visible emissions from Tb^3+^ ions are located at the most sensitive wavelength region of commercial CCD (Charged Coupled Device), silicon sensors, and other Si‐based devices, therefore Tb‐doped luminescence materials can be used for efficient spectral converters for realizing UV detection based on silicon‐based detectors.^[^ ^34^ ^]^

Herein, we have reported a new tape of Tb^3+^ doped GC obtained by a melt‐quenching method. The precipitated crystals were proved by XRD (X‐ray diffraction) and analyzed by combining the Raman spectra and TEM (Transmission electron microscopy) observations. The optical properties as well as the photoelectric properties of devices based on the developed glass and GC are compared, and the results suggest that GC exhibits much improved optical performance. The photodetector device fabricated using the optimized GC exhibits a significant photoelectric response at wavelength down to 188 nm, with a wide response range. The current research results indicate that the Tb‐doped oxyfluoride GC we designed is a promising candidate material for UV photodetectors, and the results of this study are of great significance for the development of solar‐blind UV photodetectors.

## 2 Results and Discussion

### 2.1 Design of the Photodetector

**Scheme 1:**
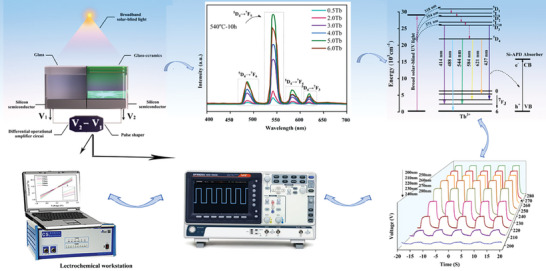
Schematic diagram illustration of the design of the photo‐detection system for broadband solar‐blind UV light enabled by the Tb^3+^‐doped GC.

The structure of the broadband solar‐blind UV photodetector by using the developed GC as the spectral converter is shown in **Scheme** **1**. The GC as the spectral converter is covered onto a silicon photo‐resistor. The operating mechanism of our detector is based on the measurement of the UV‐induced photovoltage difference between the two silicon photoresistors which are covered, respectively, with the Tb^3+^ doped GC, and a undoped reference glass. In our experiment, an electrochemical workstation and a digital oscilloscope were used to record the generated photovoltage. By UV light irradiation, the UV photons are effectively converted into visible light by the photoluminescence process of the 5% Tb^3+^ doped GC. As shown in the energy level diagram, upon UV excitation Tb^3+^ ions are promoted from the ground state to a higher excited state. Subsequently, Tb^3+^ ions are relaxed to ^5^D_3,4_ by nonradiative transition, and then to ^7^F_J_ (J = 3,4,5,6), leading to the emission of visible light photons. Finally, the visible light radiation is absorbed by the Si‐based photo‐resistor, resulting in the photocurrent, which is captured by the CS350 electrochemical workstation.

### 2.2 Structural and Morphological Characteristics

**Figure 1:**
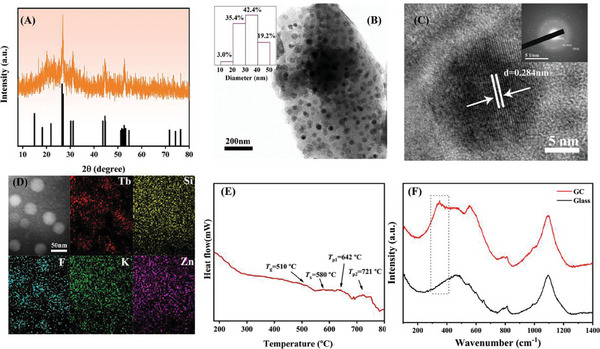
A) XRD pattern of the 5% Tb^3+^ doped GC; B) Transmission electron microscopy (TEM) images of 5% Tb^3+^ doped GC, the inset is a statistical diagram of the size distribution for nanoparticles in GC; C) High‐resolution TEM (HR‐TEM) images and SAED pattern of the 5% Tb^3+^ doped GC; (D) TEM 2D mapping images for Tb, Si, F, K and Zn elements; E) DSC curve of precursor glass doped with 5% Tb^3+^ ions; F) Raman spectra of the precursor glass and the corresponding GC doped with 5% Tb^3+^ ions.

The crystal structure of the sample was analyzed by X‐ray diffraction (XRD). The XRD result of 5.0% Tb^3+^ doped GC is shown in **Figure** **1**A, and the standard card of KTb_2_F_7_ crystal (PDF#32‐0849) is provided as a reference. It can be seen that all the diffraction peaks perfectly match the standard card of KTb_2_F_7_ crystal (PDF#32‐0849), indicating that only KTb_2_F_7_ nanocrystals are precipitated and no other secondary crystalline phase is precipitated. Interestingly, the intensities of the diffraction peaks of KTb_2_F_7_ increase obviously when Tb^3+^ concentration is increased from 0.5 to 6.0 mol% as presented in Figure S1 (Supporting Information). Accordingly, the crystallization in our designed GCs is completely governed by the doping of activator Tb^3+^ ions. This crystallization is based on a novel mechanism.

The schematic diagram of crystallization mechanisms for traditional GCs and our GCs is presented in Figure S2 (Supporting Information). For the traditional oxy‐fluoride GCs, the crystallization is governed by the glass host, and a large number of fluoride crystals are precipitated in the GCs. The rare earth (RE) activator ions were expected to enter these fluoride crystal structures via a cationic substitution process. Actually, the incorporation of RE activators into the precipitated crystals was uncontrollable in the traditional oxyfluoride GCs. Moreover, a lot of remaining crystals not occupied by RE activator ions have no contribution to the enhancement of luminescence but at the same time trigger severe issues of optical scattering and low optical transmittance in glass. In our GCs, the crystallization of KTb_2_F_7_ is completely governed by a small number of the activator ions (Tb^3^) themselves that are part of the crystal. Tb^3+^ ions were spontaneously incorporated into fluoride crystals during the crystallization process of GCs and the incorporation of RE into crystal structures is controllable. Therefore, the RE activator‐governed crystallization mechanism makes our GC a promising material possessing more efficient luminescence as well as higher transmittance as compared to the traditional oxyfluoride GCs.

As shown in Figure 1B,C, the microstructure characteristics of the 5% Tb^3+^ ions doped GC sample were studied by transmission electron microscopy (TEM), high‐resolution transmission electron microscopy (HR‐TEM), and selected area electron diffraction (SAED). It can be seen from Figure 1B that the nanoparticles are distributed within the glass matrix with diameters ranging from 10 to 50 nm and the sizes of most nanoparticles are in the range of 30–50 nm. According to the HR‐TEM image given in Figure 1C, the lattice fringes are clear, and the lattice spacing can be directly measured to be 0.284 nm, which matches the (2 0 2) crystal plane of the KTb_2_F_7_ crystals. The diffraction ring in the selected area electron diffraction (SAED) pattern in the inset of Figure 1C can be indexed by the (0 0 2), (−3 1 3), and (5 1 3) crystal surface reflections of KTb_2_F_7_ nanocrystals (PDF # 32–0849), which supports the results of XRD in Figure 1A. In addition, the SAED pattern shows some highly dispersed dots and rings with different brightness, which suggests that the GC sample is characterized by a polycrystalline structure.

The elemental mapping patterns (Figure 1D) reveal that the distribution of Tb, F, and K elements matches well with that of the location of the precipitated nanocrystals. In addition, the distribution profiles of Si and Zn elements are different from those of Tb, F, and K elements in the nanocrystal particles. These results clearly evidence that only KTb_2_F_7_ nanocrystals are precipitated in the GCs. Moreover, the high contrast of the distribution profile for Tb elements indicates that most Tb elements are distributed in the crystals in the GC.

Figure 1E shows the DSC curve of the precursor glass recorded at a heating rate of 1 °C min^−1^. The glass transition temperature *T*_g_ is located at around 510 °C and the onset temperature of crystallization *T*_x_ is around 580 °C. The first crystallization peak is located at 642 °C, which is due to the precipitation of the KTb_2_F_7_ crystalline phase. The second crystallization peak is at 721 °C. At this temperature, the glass matrix will crystallize in bulk and the glass will crystallize completely. When the heat‐treatment temperature is lower than the first crystallization temperature, precipitation of the KTb_2_F_7_ crystals from the glass occurs. Therefore, by heat treatment of the precursor glass at 540 °C, transparent GC was prepared, which contains a single KTb_2_F_7_ crystalline phase.^[^ ^35^ ^]^

To further confirm the existence of KTb_2_F_7_ crystal in GC ceramics and conduct Raman spectroscopy analysis was conducted for the glass and GC samples. As shown in Figure 1F, the main bands at around 500, 800, and 1100 cm^−1^ attributed to the characteristic vibrational modes of bridging silicon‐oxygen bonds are observed in the Raman spectra of glass and GC samples. Moreover, a new peak at around 349 cm^−1^ is observed in the Raman spectra of GC as shown in the dashed portion of the image, for which the intensity increases monotonously when the doping concentration is increased from 0.5 to 5.0 mol% as more KTb_2_F_7_ crystals are precipitated in the GCs (Figure S3, Supporting Information). The sharp peak at 349 cm^−1^ is close to the positions of Raman bands of typical fluoride crystals.^[^ ^36^ ^]^ As confirmed by the XRD results in Figure S1 (Supporting Information), KTb_2_F_7_ crystals are precipitated in the Tb^3+^ doped GCs. These results indicate that the Raman peak around 349 cm^−1^ originated from the KTb_2_F_7_ crystals. Therefore, the Raman spectra once again confirm that KTb_2_F_7_ crystals were precipitated in the GC sample.^[^ ^36^ ^]^

### 2.3 Optical Properties

**Figure 2:**
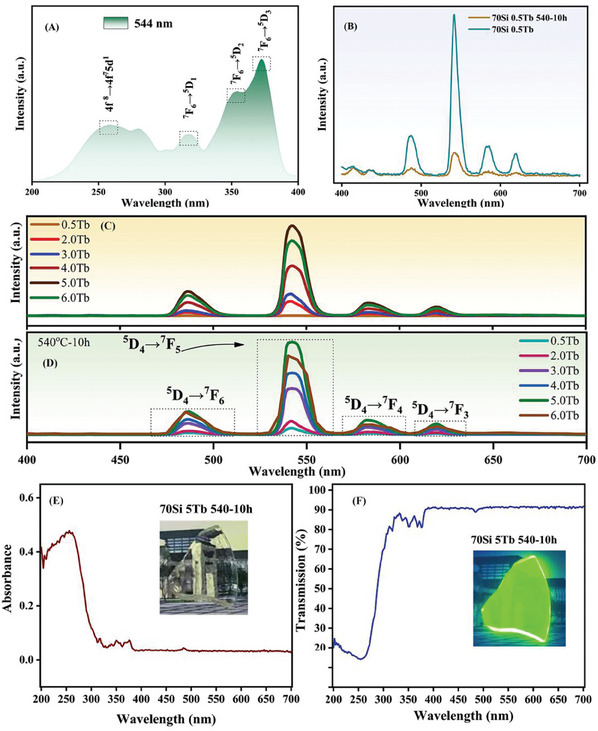
A) Excitation spectrum of the 5% Tb^3+^ doped GC; B–D) Photoluminescence spectra excited at 371 nm; E) Absorption spectra of the GC sample; Natural light irradiation. F) Transmission spectra of the GC sample; UV light irradiation.

The optical properties of the prepared GC were studied by excitation spectra, photoluminescence spectra, absorption, and transmission spectra. **Figure** **2**B–D shows the photoluminescence spectra recorded at 371 nm excitation. Figure 2B shows the comparison of photoluminescence spectra of the glass and GC doped with 5% Tb^3+^ ions. It can be seen that the luminescence intensity of GC is 5.6 times higher than that of precursor glass. Moreover, the lifetime of Tb^3+^ emission also increases from 2.25 to 2.40 ms after the heat treatment (Figure S4, Supporting Information). After the heat treatment, KTb_2_F_7_ crystals are precipitated from the glass matrix and Tb^3+^ ions enter the fluoride crystal structures (proved by the TEM mapping image), which exhibit a low phonon energy that could suppress non‐radiative transitions. Thus, the emission intensity and lifetime of Tb^3+^ are increased via the precipitation of KTb_2_F_7_ crystals after the heat treatment. Figure 2C,D shows the photoluminescence spectra of precursor glass and GC. It can be seen from Figure (Figure 2C,D) that the luminescence intensity of the 5% Tb^3+^ doped glass and GC is the strongest before and after heat treatment. Additionally, Figure S5–S7 (Supporting Information) reveal that the GC heat‐treated at 540 °C for 10 h exhibits intense emission and high transmittance. Therefore, we use the 5% Tb^3+^ ions doped GC heat treated at 540 °C for 10 h for further studies. Furthermore, the emission spectrum and quantum yield value of the traditional Tb^3+^ doped NaYF_4_ GC, a well‐established GC for efficient luminescence of RE ions, are also presented in Figures S8 and S9 (Supporting Information) to compare with those of our GCs. It is found that the emission intensity and quantum yield value of KTb_2_F_7_ GC are both higher than those of the NaYF_4_ GC. This is ascribed to the controllable incorporation of Tb^3+^ into the fluoride crystals due to the RE activator‐governed crystallization in our designed GCs.

Figure 2A shows the excitation spectrum of 5% Tb^3+^ doped GC. We can see that there are four main excitation peaks at 259, 318, 354, and 371 nm, and the strongest excitation peak is at 371 nm. A broad excitation peak appears at 259 nm, which is caused by the 4f^8^‐4f^7^5d^1^ transition of Tb^3+^ ions.^[^ ^37^ ^]^ The three excitation peaks at 318 nm, 354, and, 371 nm can be assigned to the transitions of Tb^3+^ ions from ^7^F_6_ to ^5^D_1_ (318 nm), ^5^D_2_ (354 nm), and ^5^D_3_ (371 nm), respectively.^[^ ^35^ ^]^ In Figure 2D, there are four obvious emission peaks in the photoluminescence spectra of GC samples, which are located at 488 nm (^5^D_4_‐^7^F_6_), 544 nm (^5^D_4_‐^7^F_5_), 584 nm (^5^D_4_‐^7^F_4_), and 621 nm (^5^D_4_‐^7^F_3_).^[^ ^38^, ^39^ ^]^ Since the strongest emission peak is located at 544 nm, the sample mainly emits green light under UV irradiation (as shown in Figure 2F).

Figure 2E,F shows the absorption and transmission spectra of the 5% Tb^3+^ doped GC. It can be seen that the GC sample exhibits a wide absorption range in solar‐blind UV light and is highly transparent in the visible spectral region. The transmittances of GCs are about 90% around 544 nm when the doping concentration increases from 0.5% to 5.0% as presented in Figure S10 (Supporting Information). More importantly, the transmittance of our designed GC is much higher than that of the traditional NaYF_4_ GC as shown in Figure S11 (Supporting Information). The illustration in Figure 2E,F shows the comparison of photos of the GC sample under natural light and ultraviolet radiation. These results confirm that the designed GC sample is highly transparent and emits high‐efficiency green light under UV excitation. In addition, it further shows that UV excitation light can be efficiently converted into visible emission, which is required by the spectral converter for UV detection.

### 2.4 Performance of the Photodetector

**Figure 3:**
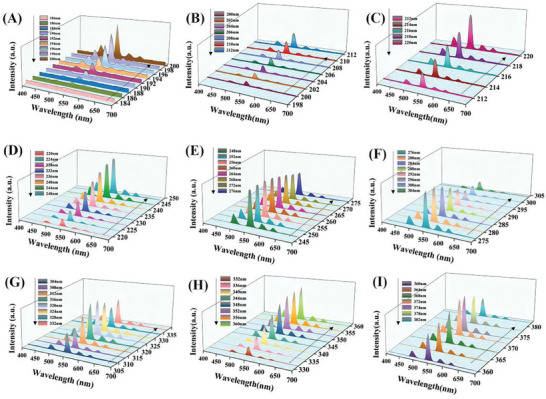
A–I) photoluminescence spectra of GC samples recorded at different excitation wavelengths.

As shown in **Figure** **3**A–I, we have studied the emission spectra of the GC sample at different excitation wavelengths in more detail. As can be seen from Figure 3A–F, under excitation at 200–305 nm, the emission intensity first increases and then decreases with the increase of excitation wavelength, and reaches the maximum at about 260 nm. At 304–332 nm, with the further increase of excitation wavelength, the emission intensity first increases then decreases, and then increases, reaching the second peak at about 316 nm. When the excitation wavelength exceeds 332 nm, the emission intensity increases significantly, reaching the third peak near 352 nm, followed by a gradual decrease. As shown in Figure 3I, the fourth peak is located at 372 nm in the range of 360–382 nm. These increasing and decreasing trends are consistence with the excitation spectrum of the GC sample given in Figure 2A. There are four obvious emission peaks in the range of 400–700 nm, corresponding to the transition of Tb^3+^ ions from the ^5^D_4_ state to the ^7^F_J_ (J = 3,4,5,6) state. As shown in scheme 1, the possible transition processes are highlighted in the energy level diagram of the Tb^3+^ ion.^[^ ^38^, ^39^ ^]^

**Figure 4:**
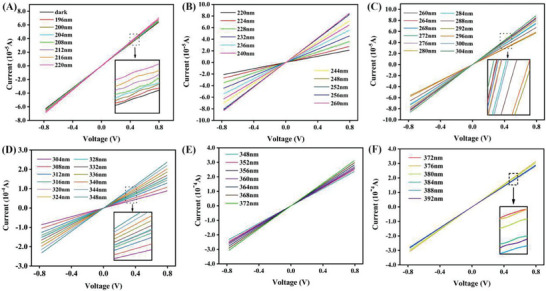
A–D) I‐V curves were recorded under excitation at different wavelengths of the UV photo‐detector fabricated by using the Tb‐doped GC as the spectral converter.

In order to study its photoelectric response, the fabricated device using a GC sample as the spectral converter is irradiated with a UV light, and the current‐voltage (I‐V) curves are recorded in the voltage range of −0.8–0.8 V. The photocurrent and photovoltage of the generated signal are displayed by an electrochemical workstation. It can be seen that the I‐V characteristic curves at different wavelengths show a linear positive correlation, which is consistent with Ohm's law. According to the I‐V characteristic curve in **Figure** **4**A, the current signal is the smallest in the dark environment. For UV irradiation at a spectral range of 196–200 nm, the curves are superimposed, indicating that the current remains almost unchanged with the increase of wavelength. Under 220–260 nm UV irradiation, the I‐V curve is separated, and the current reaches the maximum at 260 nm. As shown in Figure 4C, the current value first decreases and then increases, and when the wavelength reaches 304 nm, the current reaches the maximum value. By further increasing the UV wavelength, it can be observed from Figure 4D–F that the current value reaches its maximum at around 372 nm.

**Figure 5:**
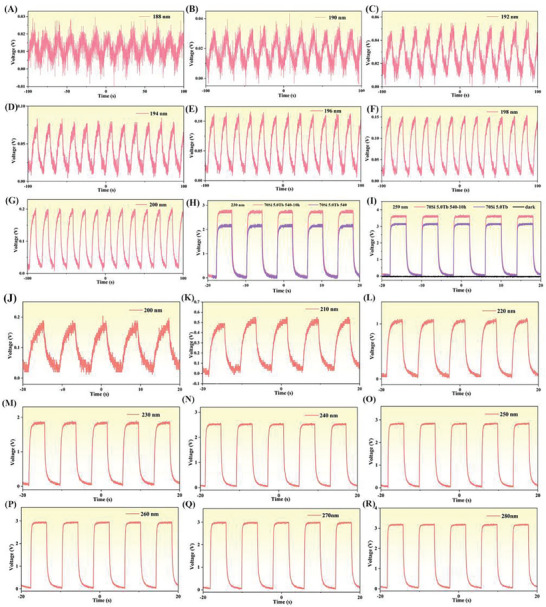
A–G) Photoelectric response of the solar‐blind ultraviolet photodetectors under 188–200 nm pulsed UV irradiation; H–I) Photoelectric response of 5% Tb^3+^ doped glass and GC recorded in a dark environment and under 230 nm and 259 nm UV irradiation; J–R) Photoelectric response of the solar‐blind UV photodetectors recorded under 200–280 nm pulsed UV irradiation.

In order to examine the temporal response of the devices, pulsed excitation light is employed. As shown in **Figure** **5** and Figure S12 (Supporting Information), repeated response to the solar‐blind UV irradiation is recorded for several on‐off cycles. According to Figure 5A–G, it is shown that a strong photoelectric response is obtained for UV wavelength down to 188 nm, with a broadband response width of over 300 nm. This result indicates that the GC material we designed can be used as a candidate material for solar‐blind UV photodetectors. From Figure 5H–I, when in the dark environment, we can see that the photovoltage of the photon capture device drops to 0 mV, indicating that the device exhibits a negligible background response. In comparison, under UV excitation, the response signal value gradually increases with increasing the excitation wavelength. As shown in Figure 5J‐R and Figure S12 (Supporting Information), the signal intensity reaches the maximum at the excitation wavelength of 371 nm. This result suggests that the UV optical signal is effectively converted into an electrical response. Furthermore, as compared with the glass, the GC shows a much stronger photoelectric response under the same experimental condition.

It is accepted that responsivity R refers to the ratio of the incident light frequency of the average photocurrent incident on the device surface, reflecting the sensitivity of the detector to incident light. It is expressed as:

(1) $$R\;=\frac{I_{\mathrm{ph}}}{P_{\mathrm{inc}}}\;=\frac{\eta q}{\mathrm{hv}}\;=\frac{\eta q\lambda }{\mathrm{hc}}\;$$

where I_ph_ is the average photogeneration current, P_inc_ is the incident light power on the device, η is the quantum efficiency of the device, hv is the corresponding photon capacity, λ is the wavelength of the incident light, q is the amount of electron charge, h is the Planck constant, and c is the speed of light in vacuum. From the equation, the responsivity of the detector device is proportional to the wavelength of the incident light. At the excitation wavelength (371 nm) where the emission intensity of Tb^3+^ is the strongest, the responsivity is relatively good. The equation also implies that light can be detected regardless of its power when it hits the photodetector.

However, for very weak light, only inherent noise can be recorded by the detector. Therefore, further determination of the noise of the detector is very important to verify the high sensitivity of the UV detector.^[^ ^40^, ^41^, ^42^, ^43^, ^44^, ^45^ ^]^ During the detection process of the ultraviolet detector device built by us, there will be noise current in its semiconductor devices:

(2) $$R\;=\frac{I_{\mathrm{ph}}}{P_{\mathrm{inc}}}\;=\frac{\eta q}{\mathrm{hv}}\;=\frac{\eta q\lambda }{\mathrm{hc}}\;$$

where *I* is the bias current of the semiconductor device, f is the test frequency, and ∆f is the band gap width of the semiconductor material. α, β, and K_1_ are all constants, and α and β≈1 under approximate conditions, which can be put into the formula to obtain the approximate noise and frequency reciprocal proportional relationship, we name its noise as 1/f noise.^[^ ^46^ ^]^ We also know that the reciprocal of frequency is equal to time, which means that noise is approximately proportional to time.^[^ ^41^ ^]^ From our results, the 5% Tb^3+^ doped samples exhibit the strongest luminescence under excitation at 371 nm, therefore the use of this sample as the converter facilitates the obtaining of the shortest response time and the fastest voltage change for the fabricated devices. Our results show that the noise is minimal at the band of 371 nm, which also confirms the characteristics of high sensitivity and high signal‐to‐noise ratio of our UV detector.

As shown in **Table** **1**, compared to other hybrid solar‐blind UV detectors recently reported in the literature, the solar‐blind UV detector developed in our work based on a simple fabrication process exhibits uncompromised performance, while demonstrating large absorption efficiency and wide response range.

**Table 1:** The distinct solar‐blind UV detectors covered in recent literature are discussed.

Type	Material	Method	Detection range	Ref.
Wide bandgap semiconductors (WBGS)	a‐GaOx	Sputtering	200–300 nm	[1]
	KNb_3_O_8_	CVD (Chemical Vapor Deposition)	230–280 nm	[2]
	ZnO–Ga_2_O_3_	CVD	200–280 nm	[3]
	α‐Ga_2_O_3_‐γ‐Al_2_O_3_	Hydrothermal treatment	250–380 nm	[5]
	AlGaN	MOCVD (Metal‐organic Chemical Vapor Deposition)	210–400 nm	[8]
	(C_5_H_6_ON)^+^(H_2_PO_4_)^−^	Facile aqueous solution	250–380 nm	[14]
	PEDOT: PSS/Ga_2_O_3_/p‐Si	MOCVD	200–250nm	[15]
	*β*‐Ga_2_O_3_	EFG (Edge‐defined Film‐fed Grown)	210–360 nm	[25]
	Graphene‐β‐Ga_2_O_3_	CVD	220–280 nm	[26]
	p‐CuZnS/n‐TiO_2_	CVD	300–400 nm	[47]
	CdMoO_4_‐ZnO	Ion sputtering	300–390 nm	[48]
	BiOCl‐TiO_2_	Impregnation	300–400 nm	[49]
Quantum Dot (QD)	ZnMgO	–	240–320 nm	[7]
	CsPbX_3_	–	280–400 nm	[50]
	MAPbCl_3_	–	340–400 nm	[51]
	ZnO QD‐Zn_2_SnO_4_	–	280–400 nm	[52]
DC (Direct Current) luminophores	Gd_2_O_3_: Eu‐PMMA	Hydrothermal	200–400 nm	[9]
Rare Earth Ions Doped Fluoroxy Glassware	Tb^3+^ ions doped GC	Melting quenching method	188–400 nm	This work

## 3 Conclusion

In summary, we have demonstrated the development of a highly sensitive broadband solar‐blind UV photodetector by using a 5% Tb^3+^ doped GC as the spectral converter. This RE‐doped oxyfluoride GC exhibits strong UV‐excited emission with a high quantum yield and a wide excitable spectral region. By combining the developed GC as the spectral converter with a silicon photo‐resistor, a stable solar‐blind UV detector based on a simple operation mechanism has been developed. Through the analysis of its photoelectric properties, it is found that the device based on the Tb^3+^ doped GC exhibits a strong photoelectric response for UV light down to 188 nm UV with a broadband response range. The results of this study demonstrate that the RE‐doped oxyfluoride GC we designed is a promising candidate material for UV photodetectors, and our results have strong implications for the development of efficient and low‐cost solar‐blind UV photodetectors.

## 4 Experimental Section

### Characterization instruments

X'Pert PRO X‐ray diffractometer (Brooke D8 ADVANCE) and energy‐dispersive spectrometer (EDS) were used for X‐ray diffraction (XRD) and elemental mapping analysis of the GC sample. At the same time, SAED and TEM images of the selected area were recorded (FEI Tecnai G2 F20). The Differential Scanning Calorimetry (DSC) curve of the precursor glass was analyzed using a synchronous thermal analyzer (Germany STA 449C). The Raman spectra of two types of glass and GC samples were studied using a high‐resolution Raman spectrometer (Horiba Evolution, France). UV‐Visible absorption spectra of the GC samples were recorded using a U3600P UV visible spectrophotometer. The photoluminescence spectra were recorded on a Zolix spectrometer, using a xenon lamp (150 W) as the excitation source. The photoelectric performance response of the photodetector was recorded on the electrochemical workstation (Wuhan CorrTest Instruments Corp., Ltd.).

### Preparation of Tb^3+^ doped 70SiO_2_‐15ZnF_2_‐15KF glass

The precursor glass containing TbF_3_ was fabricated by the melt quenching method, and the doping concentration of Tb^3+^ ions was set from 0 to 6 mol%. The molar composition of precursor glass was 70SiO_2_‐15KF‐15ZnF_2._ Before melting, a reagent‐grade stoichiometric mixture of 30 g of SiO_2_ (99.99%), ZnF_2_ (99.99%), KF (99.99%), and TbF_3_ (99.99%) was homogenized thoroughly in a ceramic mortar. The powder mixture was then placed in a covered alumina crucible and melted at 1550 °C for 30 min. Afterward, glass melt was quenched onto a cold brass plate to obtain the precursor glass. Finally, the precursor glass was heat treated at 540 °C for 10 h to obtain the GC according to the result of the DSC curve.

## Conflict of Interest

The authors declare no conflict of interest.

## Supporting information

Supporting Information

## Data Availability

The data that support the findings of this study are available in the supplementary material of this article.
