# Supplementary material for: Enabling Broadband Solar‐Blind UV Photodetection by a Rare‐Earth Doped Oxyfluoride Transparent Glass‐Ceramic
Source: Adv Sci (Weinh). 2024 Jan 15;11(12):2309433. doi: 10.1002/advs.202309433 (PMC10966569; doi:10.1002/advs.202309433)
Supplement: Supplementary file 1 — Supporting Information [file ADVS-11-2309433-s001.pdf]

ADVANCED SCIENCE
Open Access

Supporting Information

for *Adv. Sci.*, DOI 10.1002/advs.202309433

Enabling Broadband Solar-Blind UV Photodetection by a Rare-Earth Doped Oxyfluoride Transparent Glass-Ceramic

Hong Jia, Rui Zhang, Xuying Niu, Xian Zhang, Hui Zhou, Xiaofeng Liu, Zaijin Fang*, Fei Chang, Bai-Ou Guan and Jianrong Qiu*

Supporting Information

Enabling broadband solar-blind UV photodetection by a rare-earth doped oxyfluoride transparent glass-ceramic

Hong Jia[#a, f], Rui Zhang[#a], Xuying Niu[a], Xian Zhang[b], Hui Zhou[a], Xiaofeng Liu[*c], Zaijin Fang[*d], Fei Chang[e], Bai-Ou Guan[d], and Jianrong Qiu[h]

[a]College of Physics and Electronic Information & Henan Key Laboratory of Electromagnetic Transformation and Detection, Luoyang Normal University, Luoyang 471934, China.

[b]Department of Optoelectronics Science, Harbin Institute of Technology at Weihai, Weihai 264209, China.

[c]School of Materials Science and Engineering, Zhejiang University, Hangzhou 310027, China.

[d]Guangdong Provincial Key Laboratory of Optical Fiber Sensing and Communications, Institute of Photonics Technology, Jinan University, Guangzhou 511443, China..

[e]Senba Sensing Technology Co., Ltd., NanYang 473300, China.

[f]Longmen Laboratory of Luoyang, 471000, Luoyang 471934, China.

[h]College of Optical Science and Engineering, Zhejiang University, Hangzhou 310027, China.

E-mail: jiahong517@aliyun.com, xfliu@zju.edu.cn and zaijin fang@163.com

# The authors contributed equally.

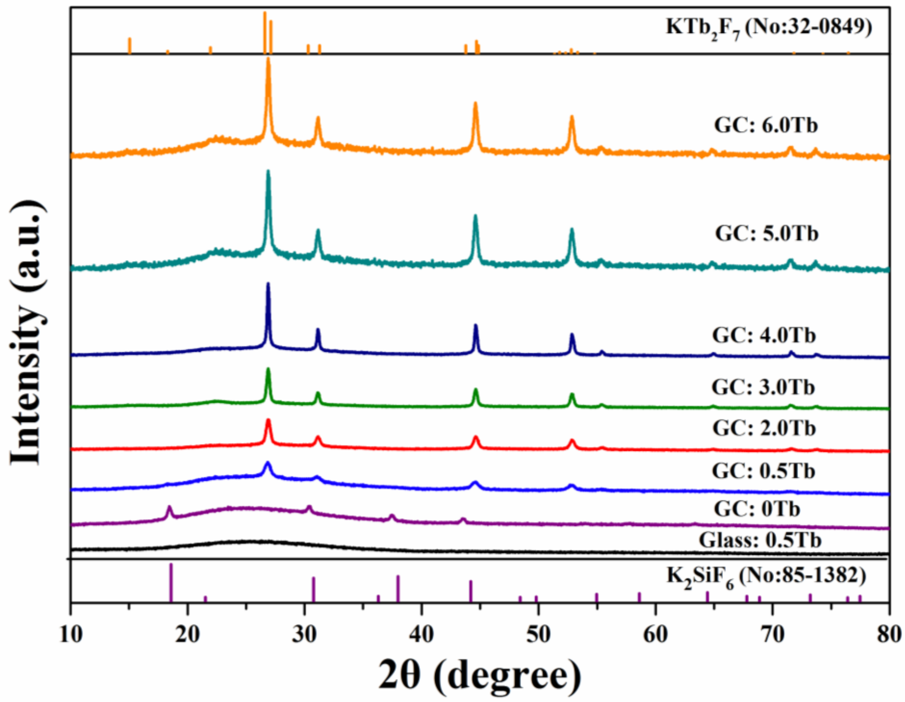

Fig. S1 XRD patterns of xTb^{3+} doped glass and GCs, and and JCPDS Cards No: 85-1382 (K_2SiF_6) and 32-0849 (KTb_2F_7). No crystal is precipitated in the as-quenched glass. After heat-treatment, only K_2SiF_6 crystals are precipitated in the no-doped GC. The crystal phase turns to KTb_2F_7 by the doping of 0.5% Tb^{3+} and the diffraction peaks increase monotonously when the doping concentration is increased from 0.5 to 6.0%.

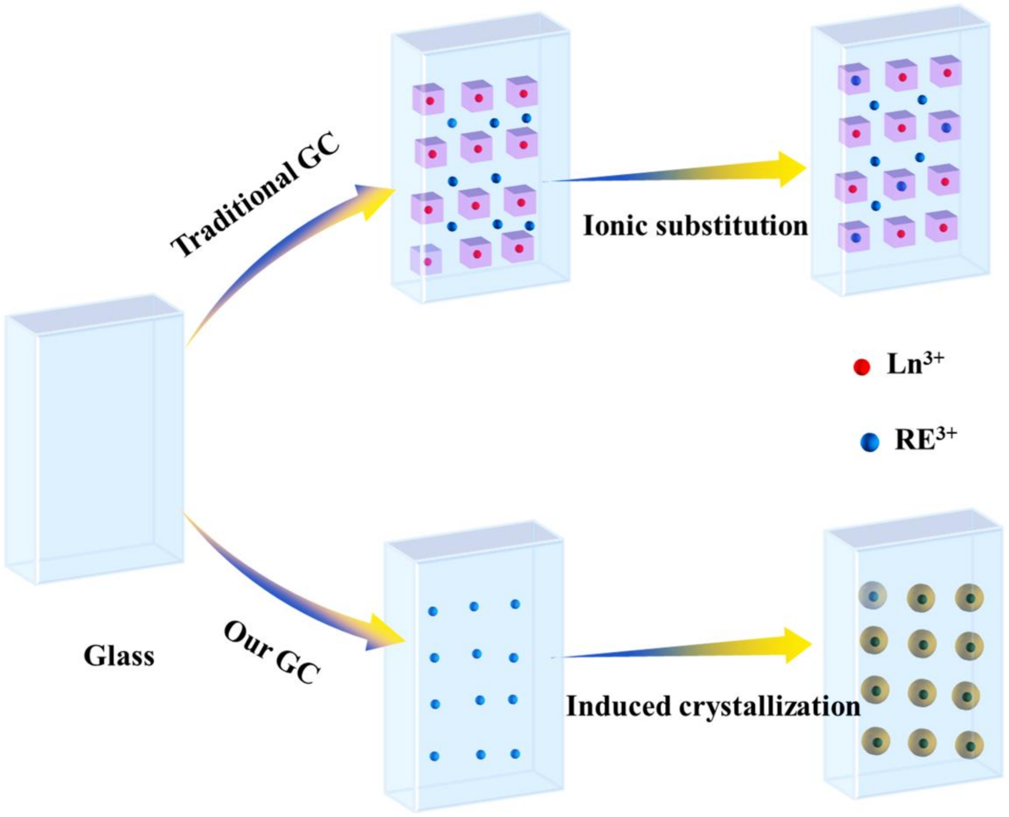

Fig. S2 Schematic diagram of crystallization mechanism for traditional GCs and our GCs. In the traditional oxy-fluoride GCs, a large number of Ln(Ln=Y, Lu, Gd, La)F_3 were added into the glass to precipitate fluoride crystals like NaLn(Ln=Y, Lu, Gd)F_4, LaF_3 and YF_3. Then rare earth (RE) activators were expected to enter these fluoride crystal structures via cationic substitution for Y^{3+}, Lu^{3+}, Gd^{3+} or La^{3+} ions. Actually, the incorporation of RE into crystal structures was uncontrollable in the traditional oxyfluoride GCs due to the due to a severe mismatch of ionic radius between RE ions and substitutable ions. A large number of fluoride crystals without the activator ions are precipitated. In our GCs, the crystallization of KTb_2F_7 is completely governed by a small number of the doped RE ions and RE ion is a part of the crystal. Tb^{3+} ions were spontaneously incorporated into fluoride crystals during the crystallization process of GCs. The quantity of crystals in GC is small and the incorporation of RE into crystal structures is controllable.

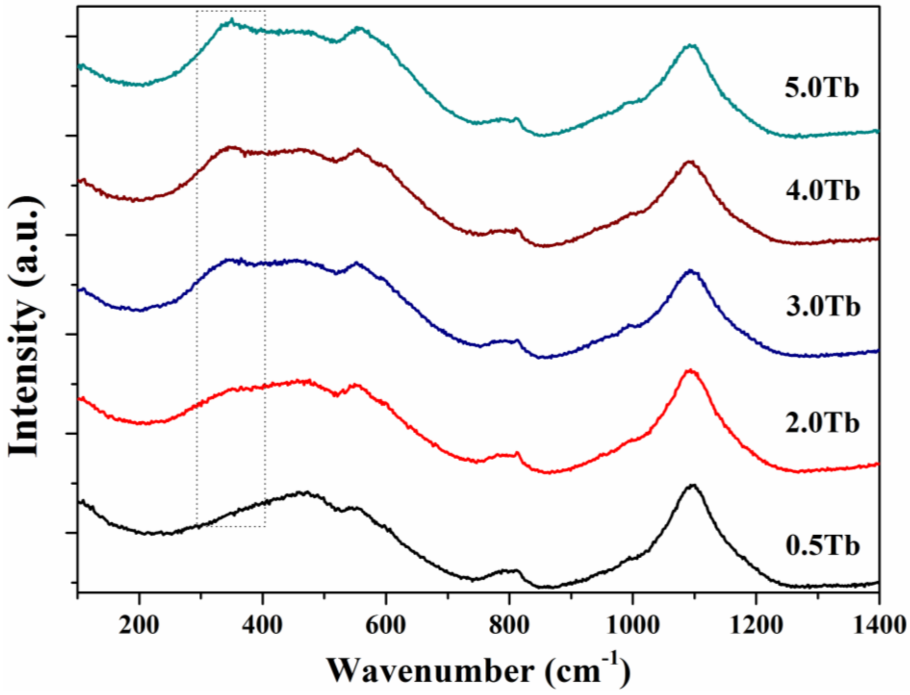

Fig. S3 Raman spectra of GCs doped with different concentration of Tb^{3+}. The intensity of the band peaking at 349 cm^{-1} increases with the increase of Tb^{3+} concentration, implying the growth in the fraction of the precipitated KTb2F7 crystals.

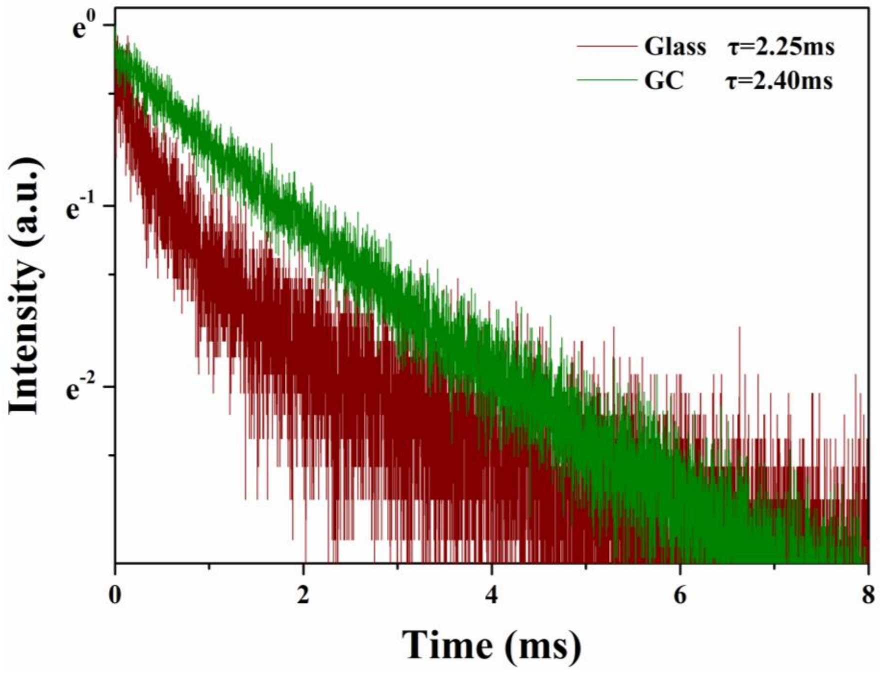

Fig. S4 Decay curves of Tb^{3+} emission at 544 nm of glass and GC heat treated at 540 °C for 10h. Excited by 371 nm light, the emission lifetime of glass monitored at

544 nm is 2.25 ms. While the lifetime in GC sample increases to 2.40 ms. These prove the incorporation of Tb^{3+} into crystal lattices.

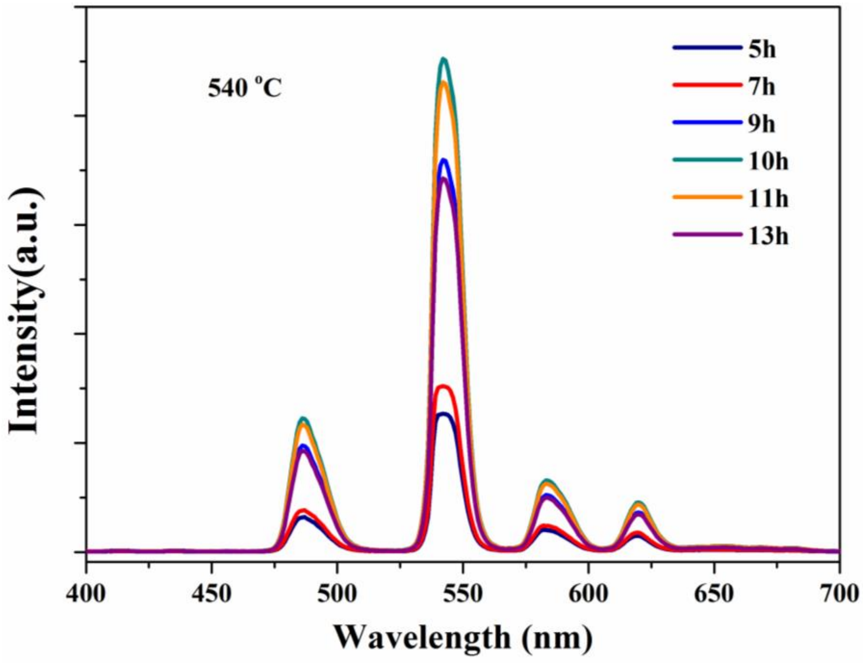

Fig. S5 Emission spectra of 5.0Tb^{3+} doped GCs fabricated by heat treatment at 540 °C for different durations. Under excitation at 371 nm, intense emissions peaks around 544 nm are observed in the spectra of GCs. The emission intensity rises as the heat treatment duration increases from 5 h to 10 h because of the increase in the number and volume of KTb_2F_7 crystals precipitated. By further increasing the heat treatment duration to 11 and 13 h, the emission intensity decreases possibly due to enhanced inter-particle coupling that leads to quenching.

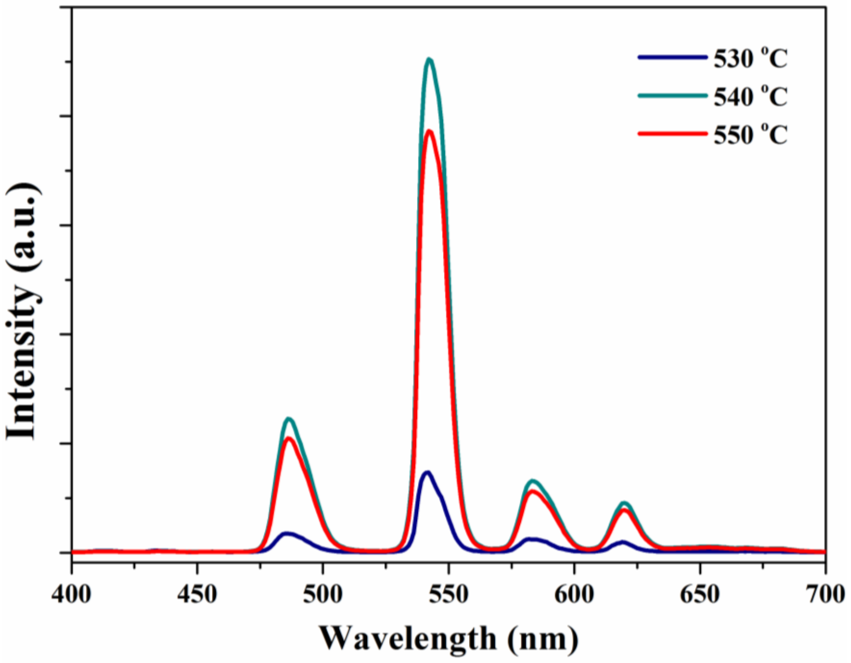

Fig. S6 Emission spectra of 5.0Tb^{3+} doped GCs fabricated by heat treatment at various temperatures. The emissions of GC heat treated at 530 ^{o}C is weak, which is ascribed to the low concentration of crystals in the sample. By rising the heat treatment temperature to 540 ^{o}C, more crystals are precipitated in the GC and the emission intensity of Tb^{3+} increases notably. However, for the GC heat treated at 550 ^{o}C, too many crystals are precipitated from the glass, which could lead to the decrease of emission caused by enhanced inter-particle coupling.

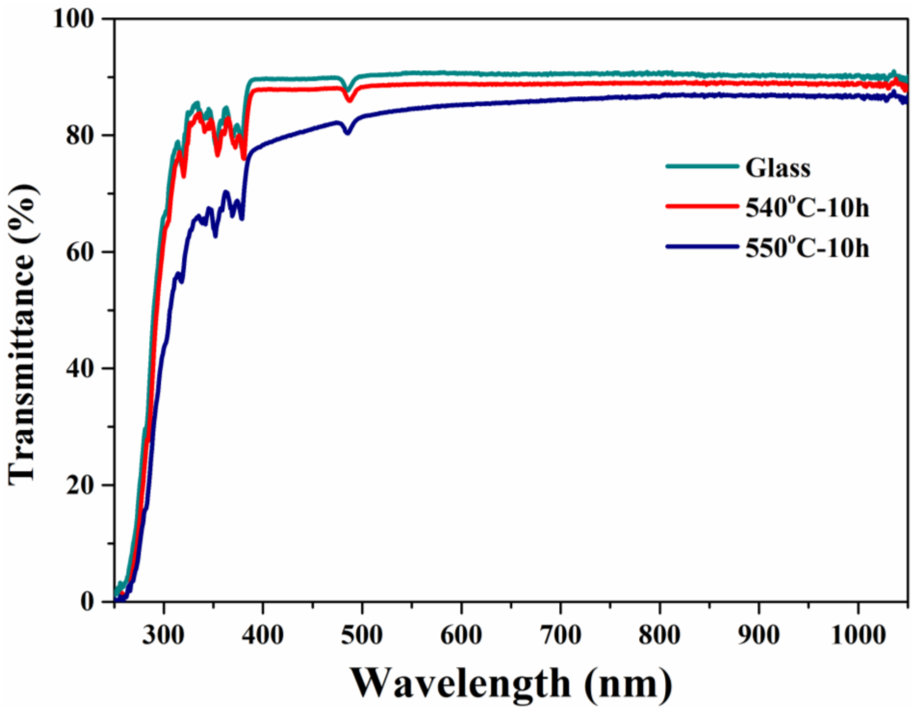

Fig. S7 Transmisson spectra of glass and GCs heat treated at different temperatures. The GC heat treated at 540 °C exhibit high transmittance (~90% at 544 nm). However, the tranmittance decreases obviously and the scattering becomes severe when the heat treated temperature rises to 550 °C due to the enlargement of crystal size.

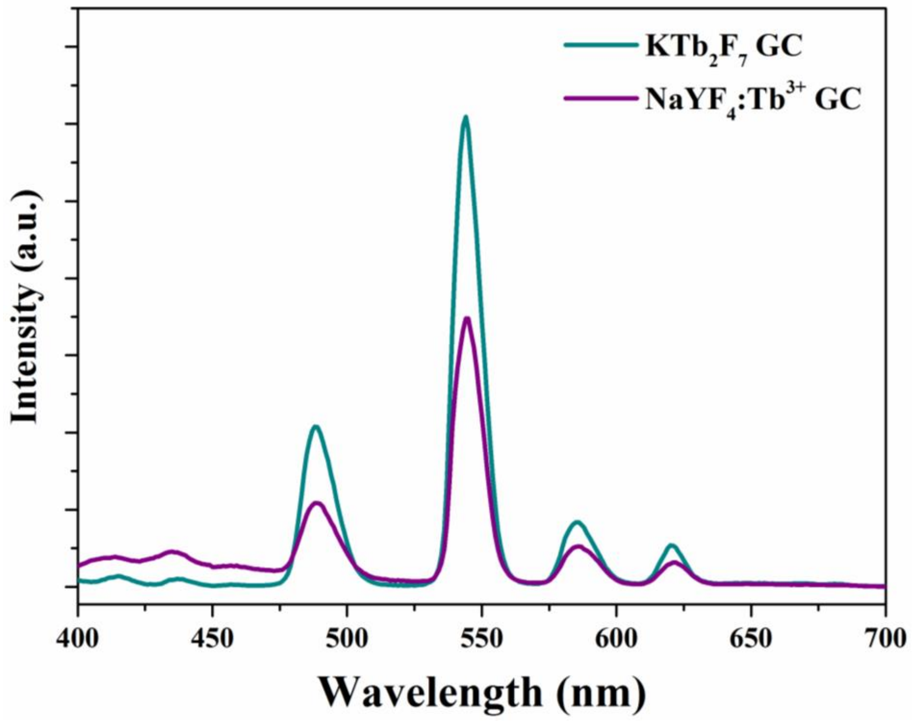

Fig. S8 Emission spectra of KTb_2F_7 GC and Tb^{3+} doped $NaYF_4$ GC. Under excitation at 371 nm, emissions of Tb^{3+} are observed in the spectra of GCs. The emission intensity of KTb_2F_7 GC is stronger than that of $NaYF_4$ GC due to the comtrollable incorporation of Tb^{3+} into fluoride crystals.

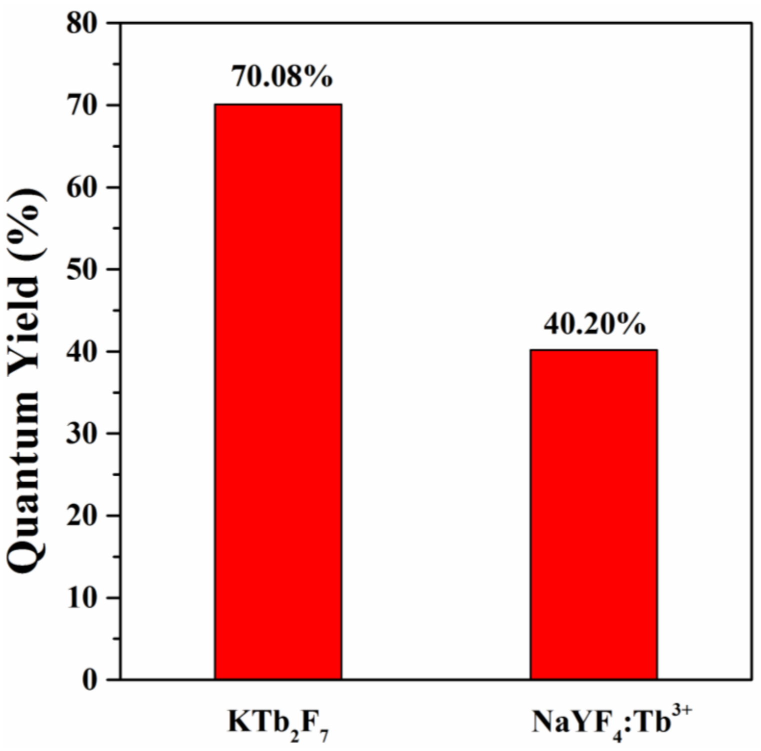

Fig. S9 Quantum yield values of Tb^{3+} emissions in KTb_2F_7 GC and Tb^{3+} doped $NaYF_4$ GC. The quantum yield vualue of Tb^{3+} emissions in KTb_2F_7 GC is 70.08%. However, the quantum yield vualue in Tb^{3+} doped $NaYF_4$ GC is 40.20%. These results indicate that our designed GC is a more efficient material for the emission of Tb^{3+}.

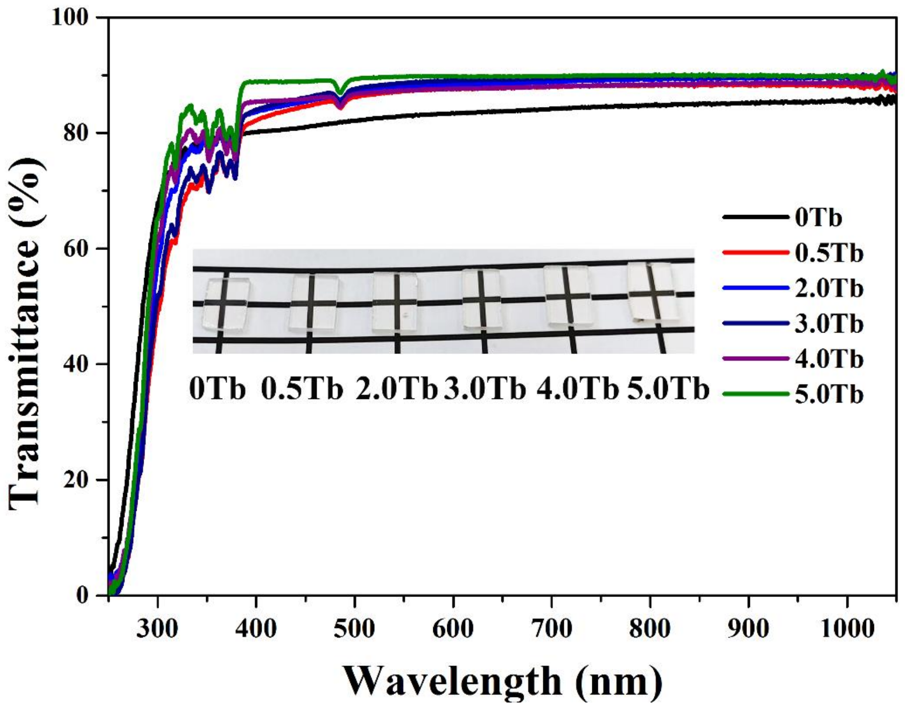

Fig. S10 Transmission spectra of GCs containing various concentration of Tb^{3+}, the inset is photos of the corresponding GC samples. As presented in Fig. S1, the diffraction peaks of Tb^{3+} doped GCs are broader than those in no-doped GC, indicating that the crystal sizes in Tb^{3+} doped GCs are smaller than in no-doped GC. As a result, the transmittance of Tb^{3+} doped GCs are all higher than that of the non-doped GC.

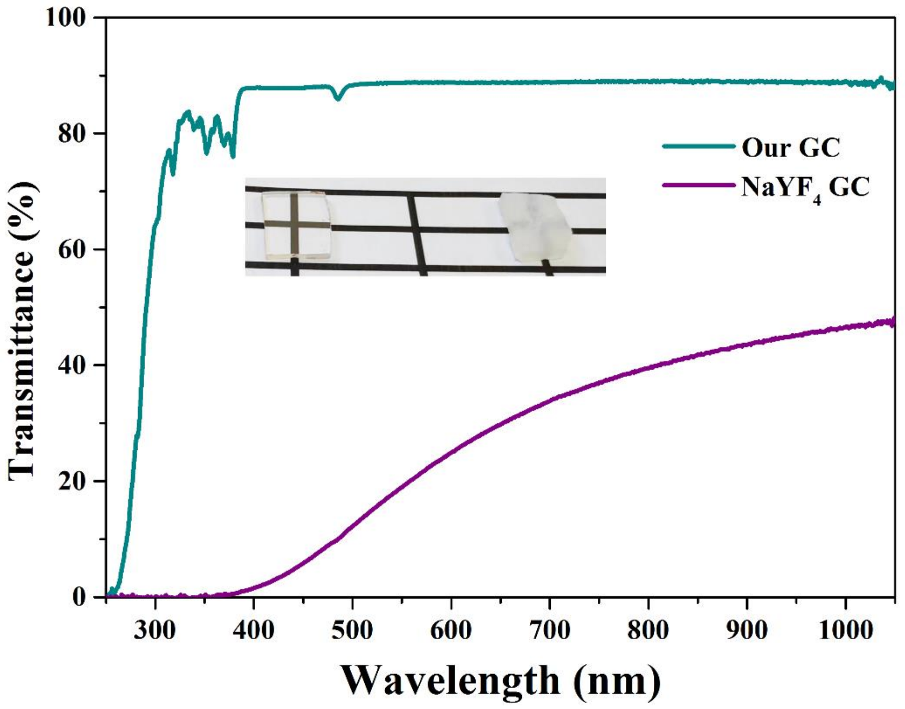

Fig. S11 Transmission spectra of our KTb_2F_7 GC and Tb^{3+} doped $NaYF_4$ GC, the inset is the photos of the GCs (left: KTb_2F_7 GC, right: $NaYF_4$ GC). The KTb_2F_7 GC possesses high tranmittance in visible region, which is much higher than the Tb^{3+} doped $NaYF_4$ GC. In the traditional $NaYF_4$ GC, a large number of crystals are precipitated in the GC, leading to severe scattering and low transmittance. The $NaYF_4$ GC is almost opaque. Therefore, our designed GC is more tranparent than the traditional $NaYF_4$ GC.

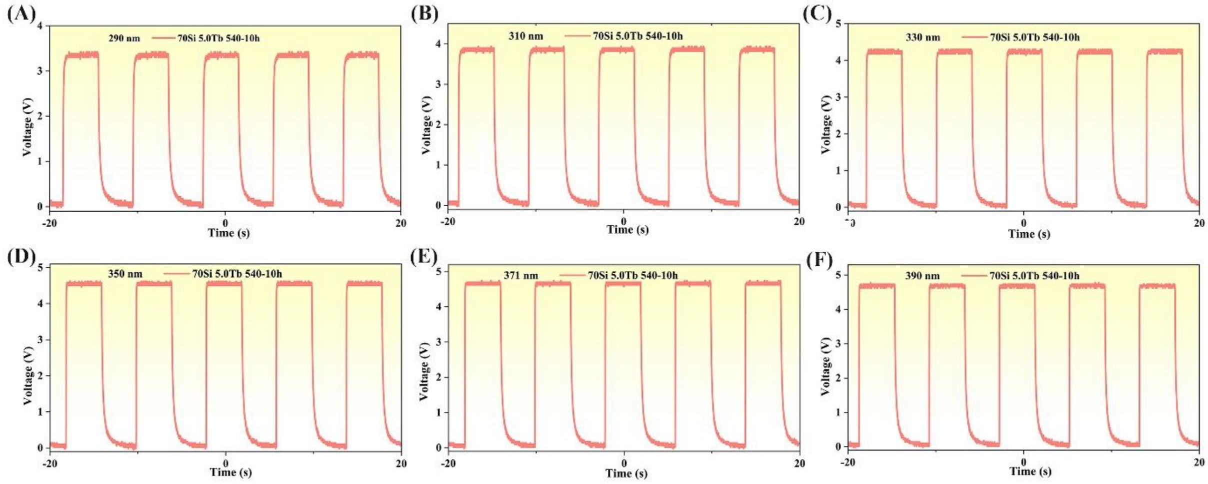

Fig. S12 Photoelectric response of solar-blind UV photodetector under 290-390 nm pulsed UV irradiation.
